# Epigenetic variation: A major player in facilitating plant fitness under changing environmental conditions

**DOI:** 10.3389/fcell.2022.1020958

**Published:** 2022-10-18

**Authors:** Vijay Rani Rajpal, Priyanka Rathore, Sahil Mehta, Nikita Wadhwa, Priyanka Yadav, Eapsa Berry, Shailendra Goel, Vishnu Bhat, Soom Nath Raina

**Affiliations:** ^1^ Department of Botany, Hansraj College, University of Delhi, Delhi, India; ^2^ Department of Botany, University of Delhi, Delhi, India; ^3^ School of Agricultural Sciences, K.R. Mangalam University, Gurugram, Haryana, India; ^4^ University School of Biotechnology, Guru Gobind Singh Indraprastha University, New Delhi, India; ^5^ Maharishi Kanad Bhawan, Delhi School of Climate Change and Sustainability, University of Delhi, Delhi, India; ^6^ Amity Institute of Biotechnology, Amity University, Noida, Uttar Pradesh, India

**Keywords:** epigenetic variability, DNA methylation, phenotypic plasticity, fitness, epigenetic memory, adaptive evolution, natural populations, climate change

## Abstract

Recent research in plant epigenetics has increased our understanding of how epigenetic variability can contribute to adaptive phenotypic plasticity in natural populations. Studies show that environmental changes induce epigenetic switches either independently or in complementation with the genetic variation. Although most of the induced epigenetic variability gets reset between generations and is short-lived, some variation becomes transgenerational and results in heritable phenotypic traits. The short-term epigenetic responses provide the first tier of transient plasticity required for local adaptations while transgenerational epigenetic changes contribute to stress memory and help the plants respond better to recurring or long-term stresses. These transgenerational epigenetic variations translate into an additional tier of diversity which results in stable epialleles. In recent years, studies have been conducted on epigenetic variation in natural populations related to various biological processes, ecological factors, communities, and habitats. With the advent of advanced NGS-based technologies, epigenetic studies targeting plants in diverse environments have increased manifold to enhance our understanding of epigenetic responses to environmental stimuli in facilitating plant fitness. Taking all points together in a frame, the present review is a compilation of present-day knowledge and understanding of the role of epigenetics and its fitness benefits in diverse ecological systems in natural populations.

## Introduction

In recent years, epigenetic modifications of chromatin material have been discovered as a major player in modulating plant fitness under challenging environmental conditions. Epigenetics refers to the study of changes in gene functions that are mitotically and/or meiotically heritable and do not involve a change in DNA sequence but are based on modifications in chromatin organization (Dupont et al., 2009). Epigenetic regulation consists of covalent modifications of DNA and histone proteins affecting the accessibility of chromatin to transcriptional machinery (Iwasaki and Paszkowski, 2014). Three major types of epigenetic marks or signatures include DNA methylation, histone modifications, and small RNAs. During DNA methylation, the methyl groups get covalently bound to cytosine nucleotides (5 mC) (Bartels et al., 2018; Gomez-Cabellos et al., 2022) and along with covalent modifications like methylation, acetylation, and phosphorylation in histone proteins regulate the local chromatin landscape (Zhu et al., 2021; Tian et al., 2022). Non-coding small RNAs such as microRNAs play a significant role in post-transcriptional regulation directing DNA methylation and chromatin remodeling at their target loci (Bossdorf et al., 2008; Robertson and Wolf, 2012; Bond and Baulcombe, 2014; Ariel and Manavella, 2021). Covalent modifications on DNA and histone proteins and small RNAs impact the condensation of chromatin and its accessibility to transcriptional machinery (Meyer, 2015). Epigenetic control mainly operates at the transcriptional level and affects the activity of genes, repetitive sequences, and transposable elements, thus providing genome integrity and helping plants survive and reproduce successfully in unpredictable environments (Kumari et al., 2022). Both genetic and epigenetic variations control gene expression independently and together.

Epigenetic mechanisms help sessile plants cope with environmental changes either by acclimation or adaptation to the changing environment. Acclimation outcomes include phenotypic responses achieved by altering gene expression and physiological attributes without incurring any genotypic changes in organisms, a phenomenon called phenotypic plasticity (Pigliucci, 2005). In other words, phenotypic plasticity is the capacity for non-genetic modifications of the phenotype. On the other hand, when phenotypic alterations are hereditary and increase the reproductive fitness of a species in relation to the environment, they are deemed to be of evolutionary adaptive significance. It has been fairly reasoned that there might be a close connection between phenotypic plasticity and adaptation, stating that plants with better plastic responses might have better adaptive ability to enhance their relative fitness, a narrative that needs to be empirically tested (Wang et al., 2021).

Environmental stress response management is quite complex and involves integrating physiological, biochemical, and genetic changes in the genome and modulating vital processes including vernalization response, genomic imprinting, and mobility of transposable elements (TEs) among others. These responses of plants could be transient in the short run or may persist over the long term. For example, Jackson and colleagues (Jackson et al., 2002) found an immediate decrease in the frequency of arbuscules formation during phosphorous sufficiency in lettuce (*Lactuca sativa*). In the longer term, only a few plant species could survive due to higher competitive abilities under resource-limiting conditions, whereas plants that control water use by reducing sap flux sensitivity survived under long-term soil moisture changes (Grossiord et al., 2018).

The most common stress-induced changes include ion toxicity, reactive oxygen species (ROS) production, redox/osmotic imbalances, nutritional disproportionality, and lipid peroxidation that bring changes in the ultrastructure of cellular and organellar membranes, chlorophyll content, photosynthetic capacities, and turgor pressure (Anamika et al., 2019; Mehta et al., 2019; Malhi et al., 2021; Singh et al., 2022). Upon perceiving these stresses, a cascade of genetic, molecular, biochemical, anatomical, physiological, and morphological changes occur in plants. While phenotypic plasticity provides plant fitness under changing environmental conditions through the spontaneous generation of epimutations (chemical changes that result in a new epi-allelic state without changing the DNA sequence), transgenerational inheritance of these epimutations induces local adaptation after many generations. These heritable epimutations encompass DNA methylation or histone modifications of a genetic locus generating epialleles (the epigenetic equivalent of genetic alleles that are stably transmitted across generations) (Robertson and Wolf, 2012; Taudt et al., 2016). These epialleles provide another layer of heritable variation to natural genetic diversity.

During the last few decades, climate change has had an unprecedented impact, which is critically categorized as a global threat to mankind in terms of agricultural and forest productivity (Leisner, 2020; Shahzad et al., 2021). Adding to risks, the average global temperature is expected to rise by 2°C by 2100 as per Intergovernmental Panel on Climate Change (IPCC) predictions (Tollefson, 2020; Pielke et al., 2022). The global climate changes have immensely impacted both the biotic and abiotic components of the environment by bringing changes in growth, multiplication, spread, virulence, and the emergence of many plant pathogens/insect pests and escalating abiotic stresses like drought, floods, heat, and cold stresses (Tito et al., 2018; Vilela et al., 2018; Juroszek et al., 2020; Skendžić et al., 2021). These environmental stresses conjointly act as a daunting obstacle that incurs fitness costs to plants. In this context, rapid phenotypic diversity that results due to alterations in epigenetic marks related to changes in chromatin architecture plays an important role. The epigenetic effects combat both short- and long-term stresses allowing natural populations to mitigate climate-change-related stresses by enhancing plant fitness (Lämke et al., 2016a).

Although epigenetic variation can be induced in the absence of genetic variability, the presence of the latter can influence the former. The genetic makeup of an individual, for instance, can affect the epigenetic marks by altering the number of cytosines available for methylation, small RNA production, availability of transposable elements, genes controlling DNA methylation enzymatic machinery, and histone-mediated chromatin modification (Feinberg and Irizarry, 2010; Robertson and Wolf, 2012). This offers further lability and advantage to the species population and adds to the ability to show transgenerational plasticity and further fuel adaptive change (Beldade et al., 2011; Robertson and Wolf, 2012). In a nutshell, genetic and epigenetic processes may complement each other or work independently, and subtle DNA polymorphisms can affect the epigenetic processes, selection, and adaptive evolution of a species. Therefore, the present review tries to compile relevant studies dealing with epigenetic processes involved in modulating plant fitness in natural populations in changing environments. The information collated will provide a platform to plan and execute in-depth studies to unravel underlying mechanisms of epigenetic regulation of plant response to environmental changes.

## Epigenetic variability generates phenotypic plasticity leading to adaptive divergence

The growth, reproduction, and survival of natural populations are largely determined by functional attributes like morphological traits, physiology, and phenology. Amidst global climatic changes that have resulted in accentuated biotic and abiotic stresses, plants have made fine adjustments and acquired additional stress mitigation strategies for their survival leading to the adaptive phenotypic divergence that depends upon natural selection, gene flow, and phenotypic plasticity (Hendry et al., 2002). Natural selection operates faster in populations with a high degree of gene flow and can fix genes that respond positively to changing environments (Charlesworth and Charlesworth, 2017; Charlesworth and Charlesworth, 2017). Adaptive divergence mechanisms in populations with a narrow genetic base, with no association with the habitats, on the other hand, indicate the involvement of epigenetic mechanisms independently or in complementation with the genetic mechanisms in facilitating adaptive evolution (Saze et al., 2012; Song et al., 2013; Amaral et al., 2020; Yi and Goodisman, 2021). Plants maximize their phenotypic plasticity either by increasing the natural variation through large population sizes or by modulating the gene expression patterns to produce altered gene functions and novel phenotypes (Wang et al., 2021). Ultimately, it is the availability of sufficient and meaningful plastic response that determines the capacity of plants to fuel reproductive success leading to fitness benefits in changing environments (Dar et al., 2022). Increased reproductive fitness serves as the basis for the survival of natural populations and their colonizing ability in the changing environments, through the amelioration of various kinds of stresses.

**FIGURE 1:**
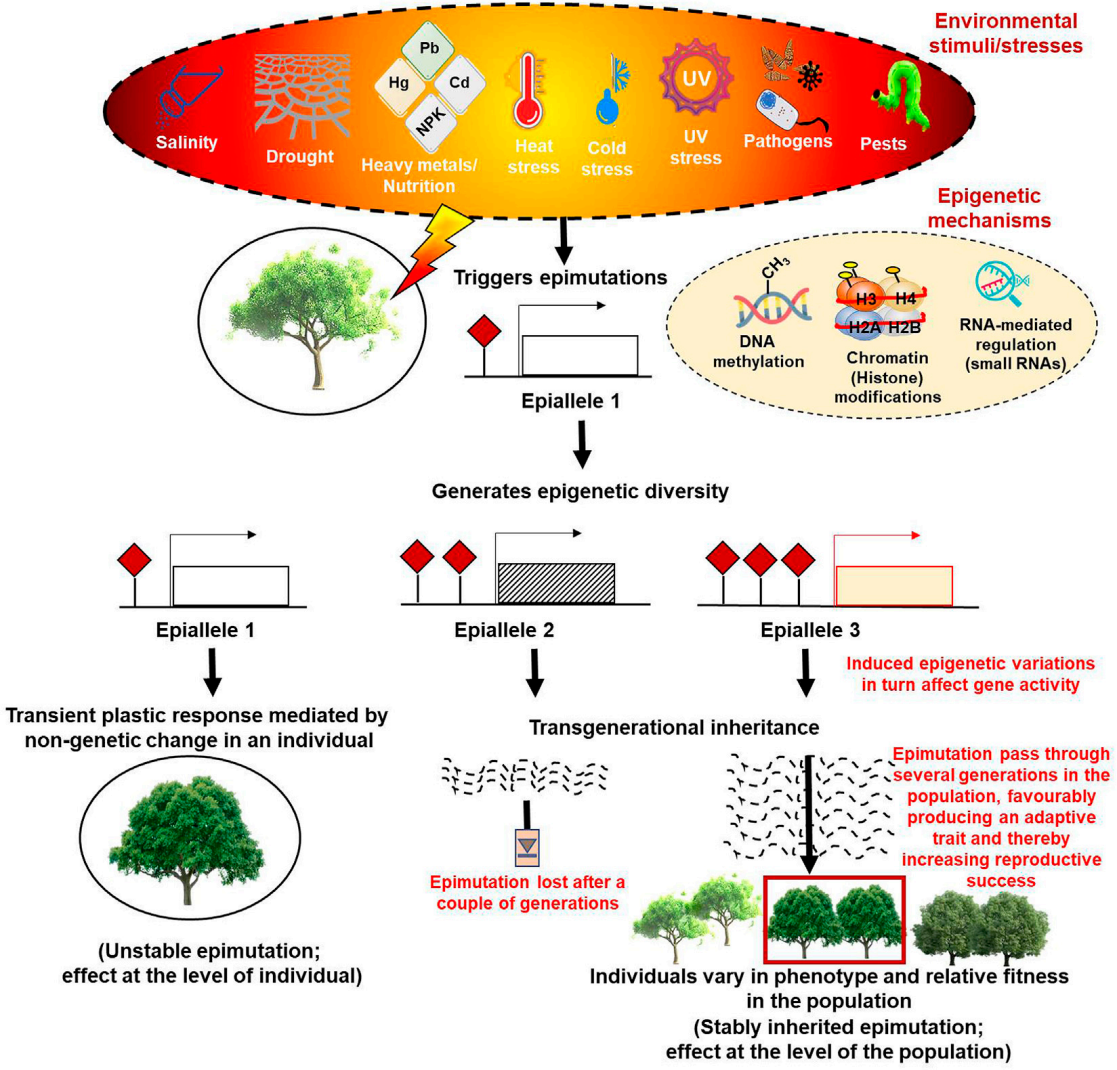
Diagrammatic representation of the epigenetic modifications based on their plastic or adaptive response to various environmental cues.

Notably, phenotypic plasticity patterns do not always follow the principles of Mendelian genetics (Foust et al., 2016; Yi and Goodisman, 2021; Guarino et al., 2022) and instead are shown to be supported largely by epigenetic mechanisms that generally originate as combinatorial effects of the interactions of genotypes with the environment (Genotype x Environment, abbreviated as GxE) (Peltier et al., 2018). The classic example showing that epigenetic mechanisms generate phenotypic variability came from studies in *Linaria vulgaris* (Cubas et al., 1999). Consequently, many more instances showing the contribution of epigenetic variability to phenotypic and functional diversity were reported (Manning et al., 2006; Martin et al., 2009; Miura et al., 2009; Herrera and Bazaga, 2013; Quadrana et al., 2014; Nicotra et al., 2015; Song et al., 2015; Ci et al., 2016; Lämke and Bäurle 2017; Schmid et al., 2018; Alonso et al., 2019; Chen et al., 2020; Baduel et al., 2021). The documentation of subtle phenotypic plasticity in homogeneous genetic populations of many self-pollinating and asexually reproducing plant species later strongly substantiated these observations (Preite et al., 2015; Wilschut et al., 2016). Although the molecular underpinnings of the causal relationship between the epigenetic mechanisms and phenotypic plasticity are yet to be clearly understood, epigenetic responses to environmental changes are shown to be mediated by the epigenome that works like a regulatory system and integrates the environmental and genetic variation to shape the inventory of available adaptive phenotypic variation (Marin et al., 2020). In this context, the epigenetic marks, especially changes in DNA methylation, can result in the induction of rapid epimutations much faster than genetic alterations and provide useful epi-allelic diversity that contributes to phenotypic plasticity. While the somatic epialleles are capable of conferring short-term responses, they provide a rapid and immediate means of negotiating the imposed stresses and ensuring the survival of populations; the long-term adaptations, on the other hand, are brought about by epialleles that show transgenerational inheritance (Figure 1) (Ashapkin et al., 2020; Yi and Goodisman, 2021).

In recent decades, emerging epigenetic processes have been highlighted as promising mechanisms to facilitate the generation of the first tier of rapid phenotypic diversity required to improve the fitness and adaptive responses in the natural populations of plants (Bräutigam et al., 2013; Amaral et al., 2020; Ashe et al., 2021). A survey of published literature shows that a large number of research investigations wherein the epigenetic route has been shown to deploy rapid phenotypic plasticity for adaptation and evolution have come from studies in model plant and crop species (Ding et al., 2018; Guarino et al., 2022; Varotto et al., 2022) which is primarily due to the availability of large genomic resources accumulated in this group of plants. The advancement in sequencing technologies and their cost effectiveness, has lately facilitated the availability of genomic resources in natural populations of non-model species as well. This data has provided useful insights into the role of epigenetic mechanisms in expanding phenotypic plasticity and adaptive responses. Even for model species, efforts have begun to compare the epigenetic and genetic heritability in field conditions.

With many published reports (Table 1, Table 2, Table 3, and Table 4), it has been convincingly shown that epigenetic processes vary among populations, affect phenotypes, stress responses, adaptability, fitness, range distribution and can also become heritable across generations. Therefore, they produce an additional, first and rapid tier of ‘epi-variability’, which individually or in combination with genetic variations have been observed to play a significant role in regulating ecological and evolutionary processes in plants (Richards et al., 2017; Baduel et al., 2021; Boquete et al., 2021). The acquired epigenetic changes allow populations to initiate the mechanisms to colonize, adapt and evolve in novel environments and the adaptations achieved get stabilized slowly by long-lasting genetic variations (Yona et al., 2015; Amaral et al., 2020). The cross-talk between the epigenetic and genetic components of variability is mediated by an interplay between changes in chromatin organization and physiological responses (Ashe et al., 2021; Boquete et al., 2021) and represents one of the most important factors contributing to the adaptive success of populations under climatic stresses.

**TABLE 1:** Instances of epigenetic regulation in plant species from different ecological communities.

Plant species (family)	Ecological community type	Inference	References
*Pinus sylvestris* (Pinaceae)	Alpine vegetation	Global DNA methylation (GDM) levels and expression of DNMT and circadian clock genes indicated that DNA methylation contributes to local adaptation	Alakärppä et al. (2018)
*Ranunculus kuepferi* (Ranunculaceae)	Alpine vegetation	The methylation patterns across two cytotypes (diploid, tetraploid) and three reproduction modes (sexual, mixed, and apomictic) were studied using MSAP. Epigenetic variation reflected an ecological gradient rather than discrete geographical differentiation	Schinkel et al. (2020)
*Wahlenbergia ceracea* (Campanulaceae)	Alpine vegetation	AFLP revealed low but significant variation between low and high-altitude seedlings. There were indications of the involvement of epigenetic mechanisms in adaptive responses to temperature stress based on MS-AFLP analysis	Nicotra et al. (2015)
*Deyeuxia angustifolia* (Poaceae)	Alpine vegetation	MSAP and AFLP analysis revealed overall low genetic diversity and epigenetic regulation suggested to be the basis for rapid adaptation to the environment	Ni et al. (2021)
*Chorispora bungeana* (Brassicaceae)	Alpine vegetation	Differential DNA methylation changes were observed due to chilling (4 °C) and freezing (-4°C) stress. Epigenetic variations are proposed as a rapid and flexible regulatory mechanism to respond to cold stresses	Song et al. (2015)
*Betula ermanii* (Betulaceae)	Alpine vegetation	A study of two habitat types, namely alpine and subalpine, from Changbai Mountain, China revealed that the alpine populations exhibited higher genetic and epigenetic diversity in the form of higher degrees of genome methylation than the subalpine populations	Wu et al. (2013)
*Rhodiola sachalinensis* (Crassulaceae)	Alpine vegetation	Four different populations *s* showed an association of altitudinal gradient and DNA methylation levels, revealing distinct genetic and epigenetic population structures in the analyzed four populations	Zhao et al. (2014)
*Prunus avium* (Rosaceae)	Temperate deciduous forests	In the five natural wild cherry populations analyzed from northern Greece, 97% of the epigenetic variability was observed within the populations. Epigenetic and genetic diversity did not differ significantly and were not significantly correlated	Avramidou et al. (2015)
*Quercus lobata* (Fagaceae)	Temperate deciduous forests	Single methylation variants (SVMs) showed significant association with four climatic variables (mainly mean temperature). SVMs located within or near the genes were known to be involved in responses to the environment	Gugger et al. (2016)
		Differential CG methylation patterns helped in adaptations to environmental changes	
*Populus nigra* cv. Italica Duroi (Salicaceae)	Temperate deciduous forests	Sixty Lombardy Poplar epigenotypes unique to each adult clone (with a single genotype), collected from thirty-seven different locations in Europe and Asia, exhibited transgenerational epigenetic variation that had an association with the bud set phenology variation suggesting the contribution of epigenetic signatures in adaptive traits	Vanden Broeck et al. (2018)
*Populus tremuloides* (Salicaceae)	Temperate deciduous forests	Significant epigenetic variability was observed between two coastal Marine stands at the Brunswick Naval Air Station. Epigenetic acclimation played an important role in the ecological success of aspen populations	Ahn et al. (2017)
*Pinus pinea* (Pinaceae)	Colder continental and Mediterranean climate communities	AFLP analysis highlighted the absence of genetic variability but MSAP identified significant epigenetic variation	Saéz-Laguna et al. (2014)
*Fragaria vesca* (Rosaceae)	Subalpine Meadows	DNA methylation pattern changes in nine study sites across three European countries: Italy, Czechia, and Norway along the climatic gradient ranging from warmest to coldest mean annual temperatures showed a correlation. With plant response to changed climate conditions. Moreover, it was found that an increase in temperature will probably be the limiting factor determining *F. vesca* survival and distribution.	Sammarco et al. (2022)
*Viola cazorlensis* (Violaceae)	Grasslands	Methylation-based epigenetic differentiation of populations was found to be associated with adaptive genetic divergence. Epigenetic diversity exceeded genetic variation	Herrera and Bazaga, (2010)
*Scabiosa columbaria* (Caprifoliaceae)	Temperate grasslands	Significant somatic and transgenerational epigenetic variability was observed between French and British populations both in the field and common gardens	Groot et al. (2018)
		The mean polymorphism observed for genetic bands was 74.8% and 64.2% in the epigenetic bands in the field and 69.4% in common garden plants	
*Vitex negundo* (Lamiaceae)	Grasslands and mixed open forests	Bayesian analysis showed a significant habitat-related genetic but not epigenetic differentiation. A significant correlation was observed between epigenetic and phenotypic variation. Epigenetic variation complemented with genetic variation to produce functional phenotypic variability for better performance in heterogeneous and diverse populations	Lele et al. (2018)

**TABLE 2:** The instances of epigenetic diversity and its relationship with environmental factors.

Plant species (family)	Environmental factor/Population description	Inference	References
*Laguncularia racemosa* (Combretaceae)	Riverside and salt marsh habitats; two populations with different exposure to salt stress	Two populations growing in different environments showed little variation with AFLP but had clear epigenetic variation as defined by MSAP.	Lira-Medeiros et al. (2010)
*Trifolium pratense* (Fabaceae)	Two populations from calcareous and oat grass meadows	Low levels of genetic and epigenetic variations. Genetic variation was influenced by the habitat-specific environment while epigenetic variation was dependent on environmental conditions	Lehmair et al. (2022)
*Lilium bosniacum* (Liliaceae)	Altitude and soil type	Two habitats, high altitude with limestone soil (1850 m) and low altitude with serpentine soil (853 m) were studied. Environment-induced epigenetic marks related to population differentiation in Lily plants differed in global methylation, presence of B chromosomes, rDNA repatterning, and chromosomal rearrangements linked to DNA demethylation that led to TE activation	Zoldoš et al. (2018)
*Populus nigra* (Salicaceae)	Soil water availability	Height and biomass were observed to be negatively correlated with global DNA methylation under drought stress. The population differentiation was very well indicated by global methylation pattern changes under drought conditions	Sow et al. (2018)
*Viola elatior* (Violaceae)	Light availability	Epigenetic population differentiation was more strongly related to habitat types than genetic differentiation	Schulz et al. (2014)
		Unmethylated and CG-methylated states of epiloci primarily contributed to population differentiation	
*Hydrocotyle vulgaris* (Araliaceae)	Flooding	Different epi-phenotypes and epigenetic differentiation between semi-submerged and submerged populations were observed. Variability was observed to be contributed by unmethylated and CHG-hemimethylated epigenetic states, specific epiloci were identified for two flood conditions	Wang et al. (2022)
*Picea abies* (Pinaceae)	Temperature and Rainfall	334 differentially methylated positions (DMPs) were identified between different populations at different temperature and rainfall levels. Differential Epigenetic methylation patterns contributed to adaptation in varied environments	Heer et al. (2018a)
*Quercus ilex* (Fagaceae)	Drought	Drought-exposed plants had a higher percentage of hypermethylated loci and reduced fully methylated loci. Changes in DNA methylation could not prevent the reduction in growth and higher mortality associated with drought	Rico et al. (2014)
*Eucalyptus grandis* × *E. urophylla* and *E. urophylla* (Myrtaceae)	Water availability	Four *E. grandis* × *E. urophylla* clones and 1 *E. urophylla* clone analyzed at two experimental sites, one with uniform precipitation and the other with a long dry season and a 4 °C higher annual average temperature, were studied. A stronger correlation was observed between the detected DNA methylation and genetic background than between DNA methylation and environment. Clone/genotype-specific DNA methylation changes at specific sites were observed	Pereira et al. (2020b)
*Hybrid Poplar genotypes*	Drought	Global methylation differences were observed within the genotypes in two of the three hybrids. Effects were more pronounced in genotypes grown for the longest durations at different habitats. Genetically identical ramets show epigenetic differences correlated to different environments	Raj et al. (2011)
*DN34, Walker and Okanese* (Salicaceae)		Environmental epigenetic differentiation was transmitted to vegetative offspring	

**TABLE 3:** Epigenetic regulation during various biological processes and functional traits development in natural plant populations.

Plant species (family)	Biological process/Phenotypic traits studied	Inference	References
*Populus x euamericana* (Salicaceae)	Winter dormancy in shoot apical meristem	Differentially methylated regions (DMRs) are identified for epigenetic memory by targeting the genes regulating the developmental response to abiotic stress under harsh environmental conditions	Gac et al. (2018)
*Populus simonii* (Salicaceae)	Photosynthesis and leaf shape development	Epigenetic regulations in the form of global methylation changes were found to correlate with developmental and functional plant traits such as leaf shape and photosynthesis	Ci et al. (2016)
*Corydalis yanhusuo* (Papaveraceae)	Sexual and asexual reproductive strategies	Significant epigenetic variation was found as compared to genetic modifications in the cultivated populations. Genetic variation was slightly higher in natural than cultivated populations. The difference was attributed to different reproductive strategies and suggested that epigenetic variation rescues response to environmental conditions in asexually cultivated plants	Chen et al. (2020)
*Dactylorhiza traunsteineri, D. ebudensis,* and *D. majalis* (Orchidaceae)	Polyploidization: Allotetraploid sibling orchid species with radical differences in ecological and geographical contexts	Genetic variation was unable to demarcate three established polyploid species with similar genetic heritage but epigenetic variation distinguished them clearly. Water availability and temperature appeared to be the key factors for this environmental allopatry in the genus	Paun et al. (2010)
*Quercus lobata* (Fagaceae)	Plant height, trichome density, leaf shape, and pathogen resistance	DNA methylation (CG/CHG/CHH) changes served as biomarkers for phenotypic variance in traits like plant height, trichome density, lobbed leaves, and powdery mildew infection	Browne et al. (2021)
*Helleborus foetidus* (Ranunculaceae)	Functional traits of leaf, flower, fruit, and seeds	The plant possessed greater epigenetic diversity than genetic variation. MSAP markers are more associated with functional traits than AFLP markers, and the epigenetic variation correlated with intra-specific functional diversity	Medrano et al. (2014)
*Hydrocotyle vulgaris* (Araliaceae)	Plant introduction: clonal herb of marshes in China	Ten populations of introduced clonal herb showed extremely low genetic diversity but high epigenetic diversity. The phenotypic diversity was found to be associated with intra-population and inter-population epigenetic diversity	Wang et al. (2020)
*Hordeum brevisubulatum* (Poaceae)	Habitat fragmentation	Higher epigenetic diversity was found in fragmented populations than in un-fragmented populations. Population epigenetic variability was found to be more sensitive than a genetic variation to habitat fragmentation in wild barley	Guo et al. (2018)

**TABLE 4:** Studies on epigenetic regulation in invasive plant species.

Plant species (family)	Inference	References
*Alternanthera philoxeroides* (Amaranthaceae)	The weed can be both terrestrial as well as aquatic. The genetic variation was found to be low but MSAP analysis showed significant epigenetic variation within and between the populations. Common garden experiments showed a correlation between epigenetic reprogramming and the reversible phenotypic response	Gao et al. (2010)
*Carpobrotus edulis* (Aizoaceae)	The plant was sampled from native and invaded areas and grown in experimental plots with changing climatic conditions. Variation was studied through MSAP. No variation was observed in response to changes in climatic variables although higher levels of DNA methylation were observed in invasive taxa as compared to the native ones	Campoy et al. (2022)
*Phragmites australis* (Poaceae)	Invasive North American populations originated from native European and Mediterranean populations. Both genetic and epigenetic mechanisms contributed to invasion success in N. America, although no epigenetic convergence was seen between native and introduced groups before genetic convergence.	Liu et al. (2018)
	Furthermore, a strong correlation was found between epigenetic and phenotypic traits than between genetic variation and the traits of twelve analyzed natural populations. Though, genetic differentiation correlated well with heterogenous habitats of the populations	Qiu et al. (2021)
*Mikania micrantha* (Asteraceae)	The analyzed 21 populations in Southern China differentiated into sub-populations with both genetic and epigenetic structures. The populations maintained an almost equally high level of transposable element-based genetic and epigenetic variation. Furthermore, the epigenetic and genetic population structure correlated with environmental variables namely, precipitation, temperature, vegetation coverage, and soil metals. The temperature variable was found to play a more important role than soil in shaping the epigenetic differentiation amongst the populations studied	Su et al. (2021)
		Shen et al. (2021)
*Spartina alterniflora* (Poaceae) and *Borrichia frutescens* (Asteraceae)	The two species were studied along a natural salt marsh environmental gradient. More epigenetic than genetic loci were correlated with habitat in both species. The relationships between genetic and epigenetic variation and habitat were observed to be species-specific	Foust et al. (2016)
*Spartina anglica* (Poaceae)	Methylation patterns (30%) between the parental and hybrids and the allopolyploid *Spartina anglica* were found to be different. The observations revealed the association between an increase in epigenetic marks and higher morphological plasticity in the invasive plant species catering to its higher ecological amplitude	Salmon et al. (2005)
*Spartina alterniflora* (Poaceae)	Significant genetic structure was observed between oil-contaminated and uncontaminated sites along with five MS-AFLP loci that correlated with oil-exposed sites. Bisulfite sequencing and epiGBS demonstrated that genetic and epigenetic variation and the expression pattern of genes correlated well with exposure to oil pollution. Both domains probably reinforced each other and contributed to the response to environmental pollution or stress	Robertson et al. (2017)
		Alvarez et al. (2020)
*Fallopia* species complex: *F. japonica, F. sachalinensis,* and *F.x bohemica* (Polygonaceae)	The analysis included five marsh sites, six roadside sites and five beach sites across eastern New York studied for habitat differentiation. Epigenetic variability was associated more with habitat differentiation in invasive taxa with poor genetic differentiation	Richards et al. (2012)
*Reynoutria* species (Polygonaceae)	The epigenetic differences observed correlated with the genetic differentiation, with a possibility of the epigenotype being dependent on the genotype. The methylation loci probably accumulated as stable epimutations and the mutations and epimutations tended to reinforce each other	Robertson et al. (2020)
*Taraxacum officianale* (Asteraceae)	In the clonal apomictic populations of invasive dandelions, range expansion along colder ecological gradients was linked to heritable DNA methylation. Furthermore, flowering time difference, an adaptive trait in colder ecological gradients was shown to be mediated by inherited DNA methylation differences	Preite et al. (2015) Wilschut et al. (2016)

## Epigenetic memory engenders quick plastic and successive adaptive responses to mitigate environmental stresses

Plants being stationary get exposed to various periodic (such as seasonal variations) or sudden (such as biotic stresses or climate factors) environmental changes. Epigenetic mechanisms help plants in generating plastic responses to stressful environments (Lämke and Bäurle 2017; Ashapkin et al., 2020; Yi and Goodisman, 2021; Guarino et al., 2022). The plants may remember the past stress(es) and use their past responses’ memory to mitigate the recurring stress(es) by generating a more pronounced and/or faster response through a phenomenon known as ‘defense priming’ or ‘stress priming’ (Lämke and Bäurle, 2017; Liu et al., 2022). It is primarily achieved by activation of stress-related genetic pathways induced by modification of the epigenetic marks cued by the environment (Bruce et al., 2007; Lämke and Bäurle, 2017; Ashapkin et al., 2020). There are two categories of genes involved in stress memory genetic pathways, namely stress-responsive genes (non-memory genes) and stress-memory genes. While the stress-responsive genes express at the same level during each stress exposure, the expression of stress-memory genes increases significantly upon repeated exposure to the same stress (Ding et al., 2012; Ashapkin et al., 2020). Table 5 collates various genes that have been observed to express in plants under stress conditions. The whole process of recognition of previous stress cues, retaining the information, and processing it to a further modified response is termed ‘stress memory’. When the stress memory is short-term and ranges from days to weeks it is referred to as ‘somatic stress memory’. However, stress memory may also become heritable and get transmitted to the offspring as ‘transgenerational stress memory’.

**TABLE 5:** List of candidate genes regulated by epigenetics in GxE interactions.

Epigenetic marks	Name	Function	References	
DNA methylation	Ac/Ds transposon	Transposon/Low-temperature stress	Steward et al. (2000)	
DNA methylation	ZmMI1 (Retrotransposon)	Cold stress	Steward et al. (2000)	
DNA methylation	HSP70 (Heat shock protein 70)	Heat stress	Aparicio et al. (2005)	
DNA methylation	PSBO1 (Oxygen-evolving enhancer protein 1–1)	Oxidative stress	Jones et al. (2006)	
DNA methylation	TAM3 (Transposon)	Low-temperature stress	Hashida et al. (2006)	
DNA methylation	NtGPDL (Glycerophosphodiesterase-like protein)	Oxidative stress	Choi and Sano, (2007)	
DNA methylation	CIPK6 (CBL-interacting serine/threonine-protein kinase 6)	Drought stress	Tripathi et al. (2009)	
DNA methylation	FLOWERING WAGENINGEN (FWA)	Flowering time	Johannes et al. (2009)	
DNA methylation	LEA46 (Late embryogenesis abundant protein 46)	Drought stress	Olvera-Carrillo et al. (2010)	
DNA methylation	Asr1	Water stress	González et al. (2011)	
DNA methylation	ONSEN (Transposon)	Heat stress	Gao et al. (2011)	
DNA methylation	POX1 (Proline dehydrogenase 1)	Drought stress	Sharma et al. (2011)	
DNA methylation	MgMYBML8 (Transcription factor)	Biotic stress	Scoville et al. (2011)	
DNA methylation	GRDP1 (Glycine-rich domain-containing protein 1)	Biotic stress	Rodríguez–Hernández et al. (2014)	
Small RNA	miR394a	Drought stress	Ni et al. (2012)	
Small RNA	miR398	Heat stress	Guan et al. (2013)	
Small RNA	miR156	Heat stress	Stief et al. (2014)	
Small RNA	miR408	Drought stress	Hajyzadeh et al. (2015)	
Histone modifications	FLOWERING LOCUS C	Flowering repressor/Vernalization	Michaels and Amasino, (1999)	
Histone modifications	FLOWERING LOCUS D	Flowering	Bastow et al. (2004)	
Histone modifications	ARP6 (Actin-related protein 6)	Heat stress	Kumar and Wigge, (2010)	
Histone modifications	ZmDREB1 and ZmCOR413 (Dehydration responsive element binding 1; cold-regulated)	Cold stress	Hu et al. (2011)	
Histone modifications	RD29A, RD20, and AtGOLS2 (Drought-inducible genes)	Drought stress	Kim et al. (2012)	
Histone modifications	ZmEXPB2 and ZmXET1	Salt stress	Li et al. (2014)	
Histone modifications	OsHAC703, OsHAG703, OsHAF701, and OsHAM701	Drought stress	Fang et al. (2014)	
Histone modifications	HSFA2 (Transcription factor)	Heat stress	Lämke et al. (2016a)	
Histone modifications	WRKY (Transcription factor)	Biotic stress	Alonso et al. (2019)	

Somatic stress memory involves short-term sustained or pronounced responses that are often accompanied by chromatin modification, histone H3K4 methylation, and reduced nucleosome occupancy (Bäurle and Trindade, 2020). These responses result in phenotypic plasticity which affects only the somatic tissues, and the phenotypic changes revert to their previous normal states when the stress conditions get over. This type of resetting is advantageous when the stress condition does not prevail in the long term because to maintain the primed response, plants need to reallocate resources that cost energy (Lämke and Bäurle, 2017). Somatic stress memory provides a specific fitness advantage under short-term stress conditions.

Most of the reports pertaining to epigenetic regulation of somatic stress memory have been derived from research investigations undertaken in the model plant *Arabidopsis* under different temperature and dehydration stress conditions. The somatic stress memory in these cases has been shown to vary from a few days to months and is observed to be associated with epigenetic changes in DNA methylation, histone proteins, and small miRNAs (Ding et al., 2012; Kim et al., 2012; Stief et al., 2014; Ashapkin et al., 2020). For instance, the trimethylation of lysine four of histone H3 (H3K4me3) has been observed in several abiotic stress-responsive genes in *Arabidopsis*. It has been observed that the accumulation of memory genes in dehydration stress regulated by H3K4me3 in the first priming stimulus is maintained throughout the memory phase (Ding et al., 2012). Similarly, accumulation of histone acetylation and activation of the *COLD RESPONSIVE* (*COR*) genes pervaded during cold stress conditions (Pavangadkar et al., 2010; Park et al., 2018). Kim et al. (2012) reported the accumulation of RD29A, RD20, and AtGOLS2 transcripts during drought stress conditions and the reduction of these transcripts to the basal level during rehydration (Table 5). This is due to the stress memory associated with acetylation of lysine nine of histone H3 (H3K9ac) and the recruitment of RNA polymerase II to these genic regions suggesting that chromatin marks play a role in transcription memory during stress conditions. Furthermore, H3K4me3 methyltransferase ARABIDOPSIS TRITHORAX1 (ATX1) which controls several genes in response to drought stress, showed abscisic acid (ABA) production that promoted drought tolerance in plants by the production of more ABA and closing of the stomata (Bäurle and Trindade, 2020).

Transcription factors are identified as key components for maintaining transcriptional memory and recruiting chromatin-regulatory proteins to their target loci. HEAT SHOCK FACTOR A2 (HSFA2) has been found to be responsible for the methylation of H3K4me in response to heat stress (Lämke et al., 2016b). Vernalization is another example of environmentally induced somatic epigenetic memory. It refers to the induction of flowering in vegetative plants upon cold exposition, a response that the plants remember as vernalization memory. This memory allows plants to remember a previous exposure to cold temperatures. For example, the FLOWERING LOCUS C gene *FLC* has been shown to accumulate H3K27 trimethylation in response to cold temperatures in *Arabidopsis*. This histone modification at the FLC locus is brought about by a Polycomb group complex recruited by VERNALIZATION INSENSITIVE 3 (*VIN3*) and VERNALIZATION 5 (*VRN5*) genes, and long non-coding RNAs (COLDAIR and COOLAIR) activated by low temperatures (Heo and Sung, 2011; Bäurle and Trindade, 2020). From the reports cited above, it has been shown that overall variation in environmental conditions can lead to local adaptation of plant populations in short term, while long-term adaptive fitness is largely derived from transgenerational effects (Lämke et al., 2016a; Lämke and Bäurle, 2017; González et al., 2018).

Transgenerational stress memory is transmitted from stressed parental generations to unstressed offspring (Bruce et al., 2007; Robertson and Wolf 2012) to help prepare future generations to mitigate environments with the same stress (es). The first instance of phenotypic variation linked to the epigenetic down-regulation of a gene (*MgMYBML8*) *was* related to the transmission of simulated herbivory (leaf damage) induced trichome production on the underside of leaves in *Mimulus guttatus.* The epimutation was transgenerational and the unstressed offspring also exhibited increased trichome production, as a response showing preparedness for future herbivory (Scoville et al., 2011). Following this, there have been many ecologically relevant intergenerational and transgenerational stress responses that are associated with epigenetic processes induced by the modification of epigenetic marks cued by the environment (Huang et al., 2010; Pecinka et al., 2010; Ding et al., 2012; Lämke et al., 2016b; González et al., 2018; Ashe et al., 2021). For instance, chromatin modification induced transcriptional activation of a repetitive element in heat-stressed *Arabidopsis thaliana* (Pecinka et al., 2010), switching from C3 to crassulacean acid metabolism (CAM) photosynthetic pathway assisted by the down-regulation of associated loci in *Mesembryanthemum crystallinum* induced by acute salt-stress (Huang et al., 2010), an increase in drought tolerance in *Oryza sativa* induced by drought-induced methylation changes (Wang et al., 2011), retention of more water by *Arabidopsis* plants subjected to multiple cycles of dehydration than the plants exposed to dehydration stress for the first time (Ding et al., 2012)*,* sustained induction and persistence of several ‘stress memory genes’ in response to heat stress correlated with different histones’ methylation (H3K4me3 and H3K4me2) (Lämke et al., 2016a) represents some stress amelioration instances mediated by epigenetic processes. The epigenetic transgenerational effects may occur across generations of both asexually (Verhoeven et al., 2010) and sexually reproducing species (González et al., 2018) and modify phenotypes of offspring generations. These changes can set a rapid pace of adaptation and microevolution in a population (Ashe et al., 2021).

In long-term stress conditions, stress memory has been observed to be inherited by successive generations mainly through the maternal parent (Wibowo et al., 2016). Expression of DNA glycosylase DEMETER (DME) in the male gametes inhibits paternal inheritance of methylation marks as they are removed by DEMETER in the absence of the hyperosmotic stress in *Arabidopsis* while in *dme* mutants the transmission of the stress memory through the paternal is restored in next generations (Wibowo et al., 2016; Lämke and Bäurle, 2017). In the natural populations of a mangrove species *Rhizophora mangle,* Mounger et al. (2021) observed a low genetic and high epigenetic diversity. About 25% of the epigenetic differences were observed among the offspring of the maternal trees and the inheritance of epigenetic variations among them was regulated by the stressed maternal environments.

The activation of many genetic components is involved in the induction of transgenerational epigenetic effects that combat long-term stress conditions allowing species to mitigate climate-change-related problems by enhancing plant fitness (Lämke et al., 2016b). Many genes have been reported in *Arabidopsis* that are influenced by GxE effects and regulated by epigenetics (Table 5). For example, Kim et al. (2012) reported the accumulation of RD29A, RD20, and AtGOLS2 transcripts during drought stress and the reduction of their amounts to the basal level during rehydration (Table 5). In another set of studies, novel phenotypes were induced in *Arabidopsis thaliana* and were linked to epigenomic patterns inherited (Schmid et al., 2018). Xu et al. (2021) showed retention of transgenerational epigenetic memory in *Arabidopsis thaliana.* A great deal of epigenetic regulation has also been observed through the activation of transposable elements (TEs) in *Arabidopsis*. The retrotransposon ONSEN which remains suppressed by RdDM (RNA-dependent DNA methylation) mechanism gets activated under heat stress in *Arabidopsis*. RdDM plays an important role in heat stress defense as mutations in RdDM machinery can limit the heat stress tolerance of plants by activating heat stress-responsive genes (Popova et al., 2013) that have been observed to be carried to future generations (Erdmann and Picard, 2020). Baduel and Colot (2021), also observed TEs-associated epi-variation that persisted for at least eight generations in *Arabidopsis* under drought, salt, UV, heat, and cold stress (Lang–Mladek et al., 2010; Ganguly et al., 2017).

In wild-type *Arabidopsis*, siRNAs target the methylation of ∼2.6 kb upstream region of the *HKT1* (HIGH-AFFINITY K^+^ CHANNEL 1) gene. In the mutants of small RNA biogenesis pathway genes such as the *rdr2* mutant, the methylation is reduced at CHG and CHH contexts. In a mutant of another gene involved in DNA methylation *MET1* (Methyltransferase 1), methylation is reduced in all three contexts (Baek et al., 2011). During salt stress, *met1* plants were observed to be hypersensitive while the *rdr2* mutant showed normal salt sensitivity. Therefore, non-CG methylation regulates the expression of *HKT1* in leaves, which may be essential in providing long-term adaptation of plants to salinity stress (Ashapkin et al., 2020).

To combat heavy metal stress, treatment of rice seedlings with heavy metals inhibited the shoot and root development in response to changing DNA methylation patterns of TEs and different protein-encoding genes (Cong et al., 2019; Ashapkin et al., 2020). In Hg^2+^-treated plants (F_0_ generation), changed methylation (CHG hypomethylation) was observed in most studied DNA sequences. In the progeny, stable inheritance of the modified methylation patterns was observed. These studies showed that the altered epigenetic state induced by heavy metal stress is heritable between plant generations for providing long-term adaptations. Further, Salicylic acid has been observed to induce epigenetic changes in the F_2_ progeny of dandelions (Preite et al., 2018).

The above studies indicate that epigenetic variation induced in response to climate changes can increase plant fitness and show transgenerational inheritance influencing long-term adaptation in populations. Thus, epigenetic mechanisms seem to play an important role in the survival, adaptation, and microevolution of plant species under challenging climatic conditions. However, future research efforts are required to establish the extent of stress response, phenotypic plasticity, their transgenerational capacity, and the mechanistic and ecological impact of epigenetics on plant adaptations in natural populations. Nonetheless, even if the induced epigenetic changes are not heritable, these rapid stress responses play a crucial role in generating transient fitness ensuring survival without switching on the expensive constitutive expression of stress-tolerant genes.

## The role of model plants in understanding epigenetic variation

During the last decade, a myriad of studies has signified the role of epigenetics in plant adaptations and its comparison with the innate genetic diversity in diverse taxa. These studies have compared genetic and epigenetic variation in natural populations of plants in contrasting environments (discussed in the next section). An alternate approach involves generating epigenetic variability in systems with little genetic diversity and has been adopted in model plants where the availability of detailed biological information, genetic resources, and immense genomic data allows answering complex questions. Many such studies have been conducted on model plants like *Arabidopsis thaliana*, *Oryza sativa*, and *Zea mays* (Richards et al., 2017).

Moreover, *A. thaliana* is ideally suited for studies of natural phenotypic variation and is a good experimental system to explore the mechanistic link between genetic and epigenetic variation, especially with regard to cytosine methylation (Schmitz and Ecker 2012). Epigenetic studies in *A. thaliana* have largely involved analysis of genome-wide methylation and epiRILs. One such study used demethylating agent 5-azacytidine and revealed differential responses in genotypes in terms of plant trait and plasticity suggesting that epigenetic variation contributes significantly to plant fitness in addition to genetic variability (Bossdorf et al., 2010). Furthermore, the investigators created epigenetic recombinant inbred lines (epiRILs) by crossing a ddm1 mutant of *A. thaliana* (*col-ddm1*) with its wild type resulting in lines that were homozygous for *ddm1*. These epiRILs were genetically nearly identical but differed in the pattern of DNA methylation (Latzel et al., 2013) and exhibited functional diversity having an effect similar to genetic diversity and leading to phenotypic variations. These two studies suggest that epigenetic diversity is an important component of functional biodiversity and can contribute to evolutionary adaptation by responding to selection at the community level (Richards et al., 2017). In a fitness-based study, the *A. thaliana* population subjected to selection in rapidly changing environments revealed a reduced epigenetic diversity suggesting that epigenetic variation is subjected to selection (Schmid et al., 2018). A study on Swedish accessions of *A. thaliana* revealed that CHH methylation is temperature sensitive while CpG gene body methylation was not affected by growth temperature. Moreover, the study concluded that local adaptation involves changes in the epigenetic landscape (Dubin et al., 2015). All these studies indicate that epigenetic diversity is providing an additional layer of variation that helps in local adaptation just like genetic variation when subjected to selection.

The studies in rice and maize are not as detailed as in *A. thaliana*, but some interesting comparative studies can be found on natural populations. These studies draw similar conclusions as from the *A. thaliana*-based studies. Targeted DNA methylation profiling has been carried out on 263 maize inbred lines (Xu et al., 2019). The study identified 16,000 differentially methylated regions (DMRs) that were not tagged by SNPs, suggesting that these DMRs possess unique information. The epigenetic variation has also been linked to inbreeding in maize (Han et al., 2021), showing hypermethylation of CHH genomic regions. DMRs have also been identified based on genome-wide DNA methylation profiles of 20 maize inbred lines. The study found that DMRs are heritable and are associated with genetic variation identified near transposable elements that may contribute to the variation in DNA methylation (Eichten et al., 2013). Similarly, MSAP marker genotyping was carried out on 50 accessions of japonica rice varieties to show more epigenetic and phenotypic similarities between the accessions grown at the same latitude (Zhang et al., 2022).

Besides these model/crop plants, several studies conducted on non-model systems exposed to diverse environments have revealed significant plasticity and can answer questions related to plant adaptations in varied environments (Thiebaut et al., 2019). These plants can serve as model plants for such studies. Examples of such systems can be identified in Table 1, Table 2, Table 3, and Table 4.

## Underpinning of epigenetic adaptive responses in natural populations by various biological processes and ecological factors

Traditionally, studies on ecological adaptations in natural populations have been comprehended through biological processes, namely genetic drift, mutation, and natural selection that work upon populations and the pre-existing genetic diversity therein. These processes are slow and take a long time to engender evolutionary divergence. To cope with the fast-changing climate and novel stresses, populations need rapid responses like phenotypic plasticity to provide the required reproductive success. It has been shown that stresses can modify epigenetic marks and generate a rapid epigenetic response(s) in the form of a wide range of novel and stable ecological phenotypes in just a few generations through gene expression regulation (Grativol et al., 2012; Heer et al., 2018a; Ashapkin et al., 2020; Ashe et al., 2021; Baduel et al., 2021; Boquete et al., 2021; García–García et al., 2022). Among all the known epigenetic mechanisms, DNA methylation is relatively stable with transgenerational heritability and contributes importantly to phenotypic variability under stress. In this context, it is extremely important to understand the mechanisms that generate phenotypic plasticity and convert local phenotypic adjustments into adaptive variability of ecological and evolutionary significance.

Rapid strides have been made in recent years to understand the role of various biological processes and other ecological factors in orchestrating strong epigenetic support to species in natural populations in generating phenotypic plasticity and acquiring evolutionary adaptations (Heer et al., 2018b; Paun et al., 2019; Amaral et al., 2020; Ashapkin et al., 2020; Campoy et al., 2022; Sun et al., 2022). The conclusive testament of their involvement in modulating epigenetic adaptive divergence in plants has come from studies conducted in plant resources representing genetically uniform backgrounds such as recombinant inbred lines (RILs and epi-RILs), asexually reproducing plant species (apomictic, and vegetative propagation) growing in varied habitats, and rapid adaptations shown by a single genotype of invasive plant species in different alien invaded habitats. Additionally, various other factors like pollination type and mating systems, polyploidy and hybridization, sympatry and allopatry as well as contrasting habitats of communities have been observed to influence the epigenetic dynamics of the genome in natural populations. The effect of these biological processes on the epigenetic landscape is reflected in the form of modifications in 1) reproductive mechanisms, 2) nuclear-cellular ratio, 3) copy number of genes altering the genome organization, 4) alterations in the expression of genes, and 5) alterations in phenotypes producing ecotypes (see examples in Tables 1, 2, 3, and 4). Studies have been undertaken to understand the spectrum of epigenetic variability in natural populations thriving under the influence of the above factors and to better comprehend the regulatory dynamics of adaptations. The findings have been tabulated for clarity, better readability, and comparisons (Table 1, Table 2, Table 3, and Table 4).

The magnitudes of heritable phenotypic variation in epigenetic recombinant inbred lines (epi-RILs) of *Arabidopsis thaliana* differing in DNA methylation but with little divergence in DNA sequence was compared with standard RILs that had considerable DNA sequence variation (Zhang et al., 2018). The lines were grown in the same environment and assessed for heritable variation in growth, phenology, and fitness. Results indicated that in some traits the phenotypic variation among epi-RILs was comparable to that among RILs and natural ecotypes, indicating that phenotypic variability can have an epigenetic basis.

Since the asexually reproducing plants lack meiotic recombination, their narrow genetic base demonstrates poor prospects for genetic adaptations. For this reason, asexually reproducing plants have even been considered as evolutionary dead ends (Lynch et al., 1993). Notwithstanding this consideration, however, there are many clonally reproducing species adapted to varied environments. Some of the most successful invasive plants of the world, for example, are clonally reproducing and many a times, a single genotype is observed to expand and thrive in diverse habitats successfully. Among the 468 invasive plants listed in the IUCN Global Invasive Species Database (http://www.issg.org/database), as many as 70% reproduce clonally. Some of the most successful invasive plants are found to be genetically uniform in their introduced ranges. Invasive clonal plant species like knotweeds in the United States, Europe alligator weed (*Alternanthera philoxeroides*) in China, *Spartina anglica* in Europe, and water hyacinth (*Eichhornia crassipes*) outside of South America show almost no detectable genetic diversity even after their establishment into a new environment (Salmon et al., 2005; Gao et al., 2010), which is primarily attributed to genetic bottlenecks and founder effects (Dlugosch and Parker, 2008). The expansion and adaptive success of invasive species with clonal uniformity and narrow genetic base in novel habitats is perplexing and challenges our understanding of the process of adaptation and indicates the involvement of additional mechanisms (Richards et al., 2012). For this reason, the study of ecology and evolutionary epigenetics has lately attracted a lot of attention in invasive plants (Salmon et al., 2005; Richards et al., 2008, 2012; Gao et al., 2010; Preite et al., 2015; Foust et al., 2016; Wilschut et al., 2016; Robertson et al., 2017, 2020; Liu et al., 2018; Alvarez et al., 2020; Qiu et al., 2021; Shen et al., 2021; Su et al., 2021; Campoy et al., 2022).

To elaborate on a few instances, Shen and coworkers (2021) reported the incidence of higher epigenetic diversity compared to genetic diversity in *Mikania micrantha* which contributed to its invasive success. They could also predict that under global warming, the populations are expanding northward, emphasizing the usefulness of such a study in predicting future invasions. The clonal plants collected from distinct habitat types of invasive Japanese knotweed (*Fallopia japonica*) in the northeastern United States showed habitat-specific phenotypes when grown in common environments (Richards et al., 2008). Further, the epigenetic differentiation with MSAP to habitat type was also found in these clonal genotypes that showed no AFLP variation (Richards et al., 2012) implying thereby that epigenetic variation allows a single clonal genotype to adjust its phenotype to different environments. Induction of environment-specific epigenetic modifications is suggested by observations in *Alligator* weed (*Alternanthera philoxeroides*), an invasive weed in China that reproduces clonally through stolons. MS-AFLP analysis of natural field-grown plants showed very little genetic but high epigenetic variation. Even after 10 asexual generations, some of the epigenetic differences appeared to be induced by environmental differences (Gao et al., 2010).

The aquatic invasive plant, *Ludwigia grandiflora* subsp. *hexapetala*, is classified as being harmful to European rivers. In French wet meadows, this species has shown a rapid transition from aquatic to terrestrial environments with the emergence of two distinct morphotypes over a period of 5 years. Both genetic and epigenetic mechanisms have been involved in adjustment to the new environment, and are supposed to be possible contributors to this fast terrestrial transition. Artificial hypomethylation was induced on both morphotypes to assess the role of epigenetics. The hypomethylation of the aquatic morphotype mimicked the characteristics of the terrestrial morphotype. This suggests that DNA methylation in a new adaptive genetic capacity is playing a key role in *L. grandiflora* subsp. *hexapetala* plasticity during its rapid aquatic to terrestrial transition (Genitoni et al., 2020).

The studies conducted on epigenetic regulation of adaptations in invasive taxa have been collated in Table 4 and show that plants adapt to different environments in many ways, and genomic and epigenomic variation provides the basis for plant adaptation and invasiveness. Epigenetic variation such as induction of DNA methylation is projected to be involved in response to diverse local environments, thus representing one crucial mechanism to promote invasion success.

**FIGURE 2:**
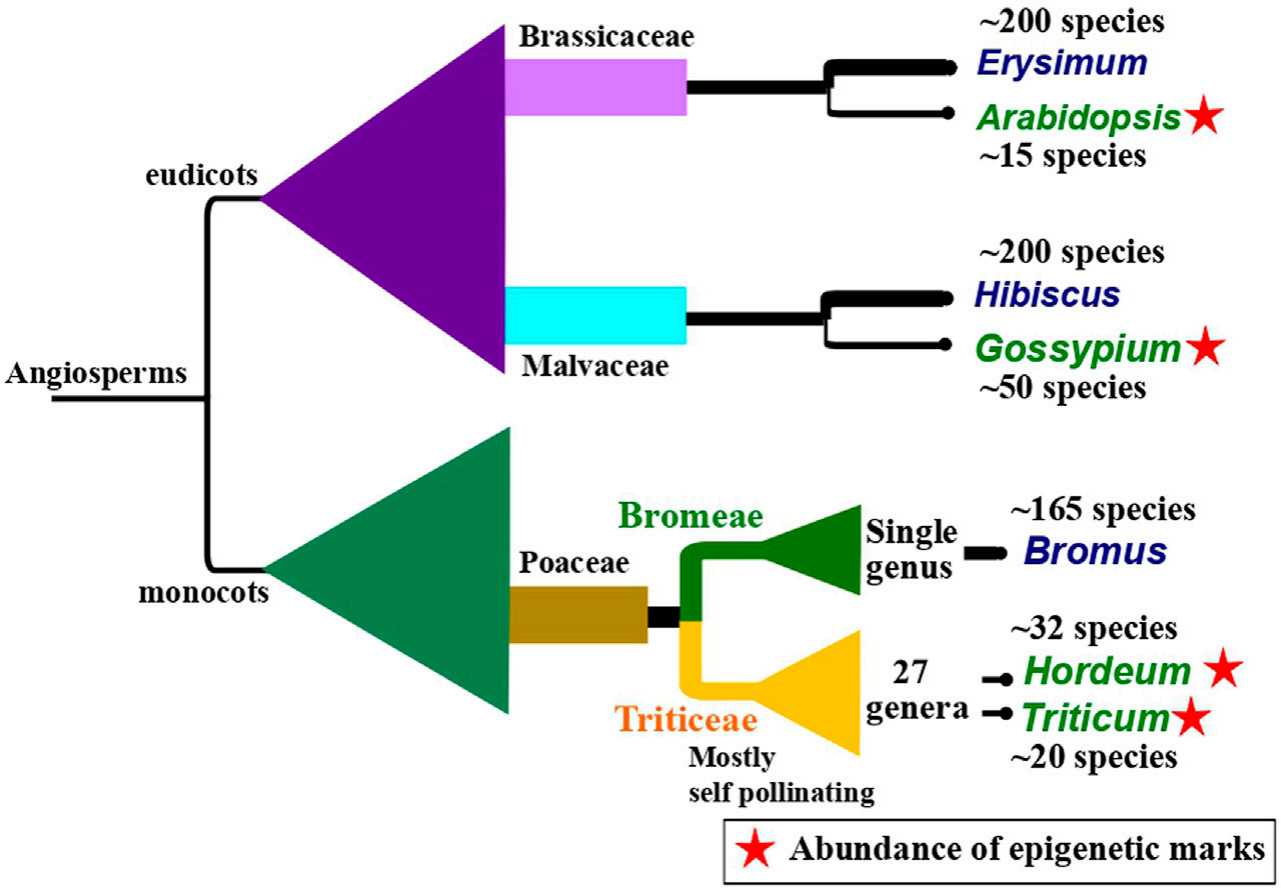
An illustration of a hypothesis of evolutionary correlation between the occurrence of self-pollinating species and the presence of abundant epigenetic marks citing three examples found across angiosperm. The illustration shows closely related pairs of sister taxa in which one is predominantly self-pollinating (taxa name in green) and the other is cross-pollinating (taxa name in blue). The predominantly self-pollinating species, namely, *Arabidopsis* (Klosinska et al., 2016), *Gossypium* (Rehman et al., 2021), *Hordeum* (Guo et al., 2018), and *Triticum* (Qiu et al., 2017) show high epigenetic marks. The phylogenetic background is drawn to show the relative systematic positions. It is generally assumed that self-pollination has evolved from cross-pollination and self-pollinating taxa have low genetic variability. Higher values in species numbers in predominantly cross-pollinating taxa might be associated with higher genetic variability in these species. The approximate species number for each taxon reported so far, has been taken from World Flora Online (World Flora Online, 2022), except for Bromus (Soreng et al., 2017).

Epigenetic inheritance has also been observed to be influenced by reproductive strategies, for instance, pollination type, and the kind and time of events leading to germline development. In plants, late germline segregation results in a longer temporal window during which epigenetic alterations caused by the environment can be integrated. A study by Preite et al. (2018) showed that alteration in DNA methylation and ncRNA expression induced by drought was inherited for two to three generations in unexposed offspring in apomictic dandelions (*Taraxacum* spp.). The study underlines the potential of long-term epigenetic inheritance in species reproducing without fertilization. Similarly, self-pollinating plants show long-term persistence of epigenetic inheritance compared to cross-pollinated plants (Wang H et al., 2009). This persistence of epigenetic inheritance might be a rescue mechanism to tackle the deprivation of genetic diversity in self-pollinating plants. Self-pollination has been thought to be derived from cross-pollination and generally leads to a loss in genetic diversity in self-pollinating plants (Stebbins, 1957). The evolutionary dynamics in self-pollinated and vegetatively reproducing plants that lack a germline have remained mystifying scientific queries for a long time. Based on the recent evidence, it is reasonable to argue that there seems to be an evolutionary correlation between the presence of self-pollination and epigenetic marks across angiosperms (Figure 2). It seems that a dearth of genetic variability is compensated by substantial epigenetic structure in a plant species (Meller et al., 2018; Kuźnicki et al., 2019; Perrin et al., 2020).

Further, the time of germline development can also affect epigenetic patterns. The mature nuclei of pollen grains that are associated with chromatin organization showed different epigenetic marks reflecting the plants’ fertilization timing strategies. For instance, DNA cytosine hypermethylation comparison of the *MROS1* gene between the vegetative and generative nuclei in pollen grains of *Silene latifolia* (Caryophyllaceae) indicated hypermethylation of DNA sequences in the vegetative nucleus during pollen grain maturation. Hypermethylation was essential for the long-term survival of the pollen until it reaches the stigma (Janousek et al., 2002). In contrast, a low level of DNA methylation was observed in the vegetative nuclei of *Quercus suber* (Fagaceae), a wind-pollinated tree, leading to active chromatin and hence a higher potential for transcription, compensating for the chromatin silencing of the generative nuclei. Further, the chromatin compaction level and transcription level of generative nuclei of *Q. suber* were greater than that of *Lilium* sps. which can be linked to differences in their pollination or fertilization processes (Ribeiro et al., 2009).

Polyploid species show increased methylated DNA, and activation of several transposons which may be essential for phenotypic divergence, adaptation, and biotic interactions (Paun et al., 2007; Fulnecek et al., 2009; Li et al., 2011; Mirouze and Paskowski, 2011; Alonso et al., 2016; Song et al., 2017; Picazo–Aragonés et al., 2020). Besides cell size, biomass, seed weight, and seed size, polyploidization is also known to affect flowering phenology between diploid and polyploid cytotypes. For example, partitioning due to ploidy differences influenced the persistence of a novel cytotype of *Anacamptis pyramidalis* that might otherwise have been out-competed by its diploid progenitor (Pegoraro et al., 2016). Niche differentiation of allopolyploid species relative to their progenitors could help the survival of invasive allopolyploid hybrids.

Hybridization and introgression also occur extensively in natural populations and contribute to diversification and speciation. Along with genomic changes, hybridization can result in epigenomic changes like activation of transposable elements (Kashkush and Khasdan, 2007; Rathore et al., 2020), small RNA changes, as well as changes in DNA methylation and histone modifications (Ha et al., 2009; Marfil et al., 2009; Kraitshtein et al., 2010; Zhao et al., 2011) which can lead to the establishment of new species *via* these stabilizing mechanisms (Feldman and Levy, 2005). In genus *Spartina*, the hybridization of an European native and an American hexaploid resulted in two genetically uniform hybrids that showed massive methylation changes in their genomes compared to their ancestors (Salmon et al., 2005). In general, allopolyploids show high epigenetic variation due to which many novel phenotypes are observed with increased species fitness (Jackson, 2017). It has also been reported that synthetic hybridization may induce chromosomal instability and hence changes in epigenetic patterns (Comai 2000; Li et al., 2010; Cara et al., 2013). Accumulating evidence also shows the significant contribution of epigenetics to heterosis.

Epigenetic regulation studies have been carried out in various ecological communities like grasslands, temperate deciduous forests, and alpine biomes (Table 1) to further understand the effect of contrasting habitats on ecological adaptations. In this context, alpine biomes represent extreme environments with harsh abiotic conditions such as extremely low temperatures, dryness, low oxygen concentration, strong ultraviolet (UV) radiation, and violent winds. Plant life in such habitats is challenging, as environmental influences can alter the conditions for development and reproduction. All these factors make alpine habitats a fragile ecosystem, ideal for studying the effect of the environment on epigenomes. Many studies in alpine plants have indicated that epigenetic modifications play an important role in the adaptive response to climatic changes and local adaptations (Wu et al., 2013; Zhao et al., 2014; Nicotra et al., 2015; Song et al., 2015; Alakärppä et al., 2018; Schinkel et al., 2020; Ni et al., 2021) (Table 1). Further, studies have also revealed interesting observations of epigenetic regulation of fitness and adaptability of populations in ecological communities like grasslands (Herrera and Bazaga, 2010), temperate deciduous forests (Avramidou et al., 2015; Gugger et al., 2016; Ahn et al., 2017; Vanden Broeck et al., 2018) and communities from colder continental and Mediterranean climates (Saéz-Laguna et al., 2014) (Table 1).

To study the effect of various environmental factors on the regulation of epigenetic dynamics, many studies have combined investigations involving field screenings of natural populations in contrasting habitats. Epigenetic variations have been enlisted in populations of selected species under ecological habitats of salt-marsh conditions against the riverside habitats or contrasting altitudes, soil, water, temperature, or rainfall conditions (Table 2). Efforts have also been done to study epigenetic regulation during various biological processes and functional traits’ development in natural plant populations (Table 3).

All the above settings have provided valuable insights into epigenetic differentiation and the role of epigenetic mechanisms in regulating trait variations. An exhaustive collation of epigenetic research conducted in natural populations in relation to various biological processes, ecological factors, communities, mating types, reproductive behaviors, and habitats will help provide a better understanding of how epigenetics can get affected by environmental stimuli and regulate phenotypic plasticity and adaptive responses during changing climates. The findings strongly propound the involvement of epigenetic regulation of phenotypic plasticity induced by environmental stimuli and substantiate the viewpoints that epigenetics plays a major role in the adaptive evolution of natural populations alone or in conjunction with genetic variability.

**FIGURE 3:**
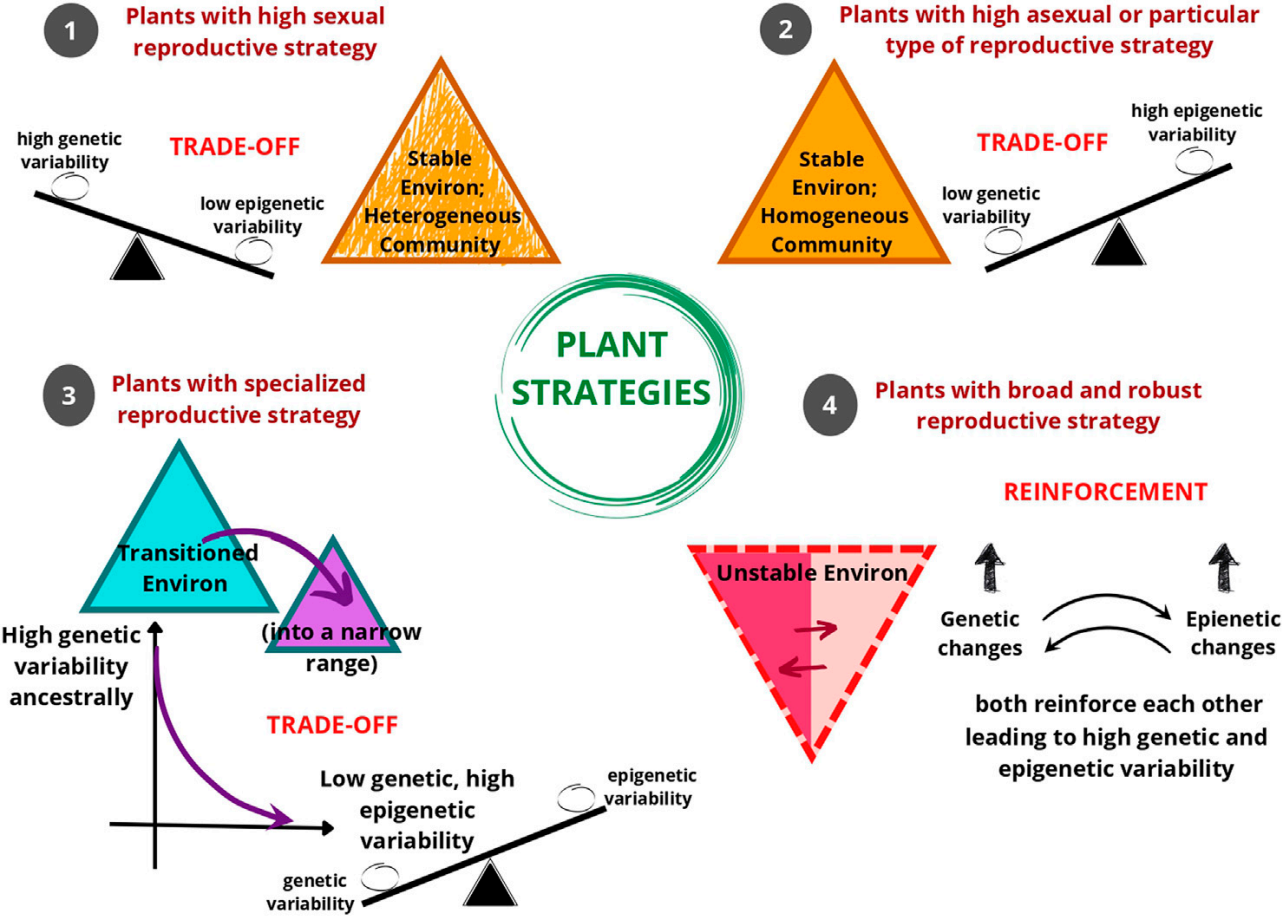
An illustration of the hypothesis that as a response to environmental conditions, different reproductive strategies of plants are associated with the coordination of genetic and epigenetic variability within the gene pool. Mostly, plant reproductive strategies show trade-offs, *i.e.* balancing of genetic and epigenetic variability. Environmental conditions (or Environ) **1**, **2**, and **3** show a trade-off between genetic and epigenetic variability in the gene pool. In Environment (**1**), the environment is stable with a heterogenous plant community, where plants generally tend to reproduce sexually. In this type of environment, the plant tends to have high genetic variability and low epigenetic variability. In Environment (**2**), the environment is stable with a homogeneous plant community, where plants generally tend to reproduce asexually or only through self-pollination. In this type of environment, the plant tends to have high epigenetic variability and low genetic variability. In Environment (**3**), when the plant transitions into an environment that is peculiar in some characteristic then the plant tends to perform specialized reproductive strategy suited for that narrow-ranged environment. In this type of environment too, there is a trend of a decrease in genetic variability and an increase in epigenetic variability. In Environment (**4**) opportunistic or invasive plants with diverse (i.e. broad) and robust reproductive responses can survive in unstable or fluctuating environments, here the genetic variability and epigenetic variability reinforce each other.

As evident, natural populations have been observed to show distinct genetic and epigenetic population structures. Even when there is low genetic variation in the population, higher epigenetic variation could be observed and *vice versa*. In some instances, epigenetic variation aligned more strongly with habitat than genetic variation indicating the link between DNA methylation patterns and the environment. We hypothesize that the interaction between the reproductive strategy of the population and its inhabited environment is crucial in determining whether the genetic and epigenetic variability exists as a balancing determiner through trade-offs or reinforce each other through the contributions of both mutations and epimutations. The plant strategizes to invest predominantly in asexual reproduction, self-pollination, or cross-pollination based on the inhabited environment that could be stable (homogenous or heterogenous) or unstable. Based on these factors, genetic and epigenetic variability finds a balance for providing a competitive edge to the plant, and finally these two pools of variability either show a trade-off or reinforcement with each other (Figure 3).

## Applications of epigenetic mechanisms in crop breeding and domestication for adaptation to the changing environment

Epigenetics plays an important role in developing strategies for crop breeding and domestication through the selection of elite crop varieties by generating more phenotypes. The induction of novel epi-alleles as plants respond to changing climates provides an additional tier of epigenetic diversity to select desirable crop traits and improve resilience. This is important as the genetic base of most of today’s crop cultivars has become narrow due to recurrent selections (Gallusci et al., 2017). The availability of sufficient variability in the gene pools is pivotal for executing meaningful and successful crop improvement programs to ameliorate various biotic and abiotic stresses arising due to the changing stressful global climates (Samantara et al., 2021; Krishnaswami et al., 2022). Stress exposure cascades a response-signaling cycle in plants that along with genetic modifications leads to many epigenetic changes routed through mechanisms such as chromatin remodeling, DNA methylation, histone modification, and non-coding RNAs (Turcotte et al., 2022). These epigenetic mechanisms have resulted in novel epialleles associated with morphological, metabolic, and developmental traits in plants (Tirnaz and Batley 2019) that can be exploited for developing new climate-resilient crop varieties to mitigate the challenges of changing environments (Springer and Schmitz, 2017; Kawakatsu and Ecker, 2019; Varotto et al., 2020; Kakoulidou et al., 2021; Guarino et al., 2022) and help in crop domestication by targeting epigenetic diversity to engineer crop plants for optimal crop production (Ding and Chen, 2018).

The epigenetic variability has shown tremendous potential for the selection of agronomic traits and QTLs in *Arabidopsis* and other important crops like rice, rapeseed, soybean, cotton, maize, and tomato. The bilateral symmetry of flower development in *Linaria cycloidea*, FWA locus involved in regulating flowering time in *Arabidopsis*, colorless non-ripening locus in tomato, vitamin E accumulation in tomato, improved anthocyanin production in apple, dwarf plant stature in rice, sex determination in melon, slower fruit ripening in mature tomato, somaclonal variation in oil palm, increased embryogenesis in pineapple, high heterosis in pigeon pea, altered oil content in oilseed rape, enhanced yield in soybean and adaptation of upland domesticated rice in response to osmotic stress among others exemplify some of the epigenetic variants showing useful traits in crops (Kakutani, 1997; Cubas et al., 1999; Manning et al., 2006; Martin et al., 2009; Miura et al., 2009; Telias et al., 2011; Zhong et al., 2013; Nonogaki, 2014; Quadrana et al., 2014; van Zanten et al., 2014; Ong-Abdullah et al., 2015; Xia et al., 2016; Ding and Chen, 2018; Luan et al., 2020; Sinha et al., 2020).

In rice, changes in DNA methylation and/or histone methylation and acetylation patterns have resulted in many epialleles linked to desired crop traits like increased panicle branching and yield (*OsSPL14*) (Miura et al., 2010), delayed flowering (SDG724 induced loss of function mutant Ivp1), increase in grain size, weight, height and yield (down- and upregulation of the genes *OsLAC* and *OsglHAT1*, respectively) (Song et al., 2015) and grain yield (*OsPCF7*) (Li et al., 2020). Likewise, while an increase in DNA methylation mitigated cold stress in tomato, heat and salinity stress in rapeseed, drought stress in faba bean and controlled photosynthetic activity in rice (Wei et al., 2017; Samantara et al., 2021), the hypomethylation of *ESP* gene regulated the improved panicle architecture in rice (Luan et al., 2019). The methylation of histone H3 lysine 36 (H3K36), acetylation of H3K9, and histone H3 lysine 27 trimethylation (H3K27me) have played key roles in bringing about many epigenetic changes in rice (Gupta and Salgotra 2022). Similarly, alterations in H3K4me2 in maize helped in mitigating abiotic and biotic stresses (Samantara et al., 2021) and histone acetylation was shown to regulate drought stress in tomato and *Arabidopsis* (González et al., 2013; Zheng et al., 2016). It has been demonstrated that histone deacetylase HDA9 plays a key role in regulating drought stress in plants (Turcotte et al., 2022). Further, miRNAs regulated the expression of the *OsSPL14* gene affecting the panicle branching and increasing the rice yield (Miura et al., 2010).

Further, a comprehensive epigenomic and functional analysis of domesticated allotetraploid cotton and their tetraploid and diploid relatives revealed differentially methylated cytosines regions that contribute to domestication traits such as flowering time and seed dormancy in cotton (Song et al., 2017). In another study, domestication was exhibited through DMRs using 45 soybean accessions. Interestingly, no association was observed with the genetic variation enriched in carbohydrate metabolic pathways (Shen et al., 2018). In rice, flowering time is crucial for seed set which is regulated by histone methyltransferase (HMTase) genes such as *SET DOMAIN GENE 724* (*SDG724*) that promotes flowering by methylating the histone H3 lysine 36 (H3K36) (Sun et al., 2012; Gupta and Salgotra, 2022). The useful epigenetic diversity in many other crops has been collated well in recent review articles (Varotto et al., 2020; Kakoulidou et al., 2021; Alfalahi et al., 2022; Gill et al., 2022; Guarino et al., 2022; Gupta and Salgotra 2022; Sharma et al., 2022; Villagómez-Aranda et al., 2022). All these studies suggest that the identification of agronomic traits regulated through epigenetics will provide new opportunities for crop improvement and domestication.

Since it has been established that the epigenetic mechanisms aid plants in stressed conditions (Chachar et al., 2022), efforts are underway to understand the mechanism of epigenetic enzymatic systems that help in recognizing, installing, and erasing the epigenetic marks. The availability of cutting-edge sequencing technologies coupled with computing methods like machine learning and deep learning is geared towards decoding the role of epigenetic regulation in crop species (Ali et al., 2022). Although many instances of spontaneous epi-allelic diversity have been documented, efforts are also underway to induce epigenetic diversity in crops. In this context, ‘Eustressors’ that involve low-dose exposures to stress factors to trigger positive responses in plants through effects on their physiology, biochemistry, genetics, and epigenetics have emerged as potent breeding tools (Villagómez-Aranda et al., 2022). This is an emerging area of research and future efforts aimed at identifying eustressors capable of generating stable epigenetic marks, understanding mechanisms leading to trans-generational memory to stimulate a priming state and their adaptability potential are essential before they can be used to induce epigenetic variability for the advancement of important agronomic traits in crop plants. In the nutshell, emerging epigenetics-based technologies highlight the prospects of novel epi-alleles to broaden the genetic base of crop plants and open vistas to use epi-variability to support future crop epi-breeding and domestication programs. Integrating epigenetic methods into traditional and modern crop breeding and domestication exercises can serve as an expanded toolbox to address the agricultural productivity challenges.

## Techniques to identify and quantify epigenetic signatures

Traditionally, the study of epigenetic changes has been based on DNA methylation profiling, methylation-sensitive amplification polymorphism (MSAP) (Waalwijk and Flavell, 1978), and bisulfite sequencing (Cokus et al., 2008; Kurdyukov and Bullock, 2016), though the advent of techniques like chromatin immunoprecipitation (ChIP), next generation sequencing (NGS), and bisulfite reduction sequencing (bsRAD) (Trucchi et al., 2016) in the recent years has changed the scenario of data generation in studying epigenetic aspects. Significant efforts have been undertaken for technological simplification to study DNA methylation changes in natural populations or species with large genomes (Chwialkowska et al., 2017). The methods used in epigenetic studies are listed in Table 6.

**TABLE 6:** Commonly used NGS-based techniques for epigenetic analysis.

Technique	Application	References
WGBS (Whole genome bisulfite sequencing)	The whole genome with all cytosine contexts can be studied	Lister and Ecker (2009)
MeDIP-seq (Methylated DNA Immunoprecipitation)	The whole genome can be studied with a small amount of initial DNA	Mohn et al. (2009)
RRBS (Reduced Representation Bisulfite Sequencing)	Can be applied to large-size genomes without a reference genomes and all cytosine contexts can be studied	David et al. (2017)
MSAP-seq (Methylation Sensitive Amplification Polymorphism Sequencing)	Can be applied for large-size genomes without a reference genomes	Chwialkowska et al. (2017)
ChIP-seq (Chromatin Immunoprecipitation Sequencing)	Can assess histone modifications; provides a genome-wide map	Diaz et al. (2017)
MS-DArT (Methylation Sensitive Diversity Array Technology)	The whole genome can be studied without a reference genomes	Pereira et al. (2020a)

Of all the methods discovered so far, MSAP remains the most widely used due to the associated ease of performance and the simplicity of the technique. It is similar to amplified fragment length polymorphism (AFLP) in procedure but uses two isoschizomers *Hpa*II and *Msp*1, that differ in their sensitivity to the methylation state of cytosine (Waalwijk and Flavell, 1978). The advent of NGS has allowed MSAP to become a high-throughput method known as methylation-sensitive amplification polymorphism sequencing (MSAP-Seq) (Chwialkowska et al., 2017). However, poor efficiency of the restriction enzymes to cut at CHH and CHG contexts than CG context is a limitation associated with this technique as this can portray an incorrect methylation status of the genome.

The bisulfite treatment is the next most widely used technology after MSAP. It is used for the detection of cytosine methylation both at specific loci and genome-wide levels. Bisulfite treatment of genomic DNA results in the deamination of unmethylated cytosines to uracil but methylated cytosines are not prone to this treatment and still read as C and can be distinguished from unmethylated cytosines (Jones, 2012). The downstream analysis after bisulfite treatment can be done by using several methods such as direct sequencing of bisulfite PCR product, sub-cloning of PCR product followed by sequencing, combined bisulfite restriction analysis (COBRA), methylation-specific PCR (MSP), and pyrosequencing (Li and Tollefsbol, 2011).

The advent of NGS has made it possible to look at DNA methylation changes at the whole genome level using whole genome bisulfite sequencing (WGBS) (Meissner et al., 2008; Lister et al., 2009). However, the required depth becomes a bottleneck for large, complex genomes, resulting in increased costs. To address this problem, a method like reduced representation bisulfite sequencing (RRBS) was developed, where an enzyme is used to target a specific portion of the genome, which often is an enzyme like *Msp*I, which targets 5′CCGG3′ sites often found in promoters (Meissner et al., 2005). RRBS allows working with large genome sizes as it constitutes genome fragmentation that reduces the complexity of genomes (Davey et al., 2011).

The methods have also been developed to target particular sequences for DNA methylation analysis such as bisulfite padlock probes (BSPP) (Diep et al., 2012). Although the bisulfite-based NGS methods have provided a single base resolution of epigenetic changes, it fails to provide information on specific cells when it comes to complex tissues. In this context, the recently developed, single-cell bisulfite sequencing (Smallwood et al., 2014) technique provides whole epigenetic insights into individual cells.

The utility of bisulfite sequencing in natural populations became possible after adapting the RADseq and GBS protocols to procure the methylation profile of reduced genome libraries for species where a reference genome is not available. Three RRBS protocols namely epiRAseq (Schield et al., 2016), bsRADseq (Trucchi et al., 2016), and epiGBS (van Gurp et al., 2016) find great utility in this context. Methylation-sensitive, MS-DArT (Pereira et al., 2020a) has emerged as another promising technique to study DNA methylation profiling. It is a genotyping technology that involves double digestion preceded by adaptor ligation and NGS. The technology is attractive and amenable to use for any species as the availability of a reference genome is not imperative, though it is useful to establish the genomic context of methylated bases.

Another technique for accurately studying DNA methylation has been through the deployment of antibodies developed against 5-methylcytidine (Mohn et al., 2009). This method is called Methylated DNA immunoprecipitation (MeDIP) which can be combined with simple PCR, microarrays, or NGS to create a powerful tool for the unbiased detection of methylated DNA. MeDIP can be combined with MRE-seq (methylation-sensitive restriction enzyme sequencing) for more accuracy in methylation studies (Maunakea et al., 2010). MRE-seq involves fragmentation of DNA using methylation-sensitive restriction enzymes followed by sequencing. Methyl-binding domain isolated genome sequencing (MBDiGs) is another technique that involves recombinant Methyl Binding Domain (MBD) and MBD2 proteins for the enrichment of methyl-rich DNA fragments (Serre et al., 2010).

Antibodies have also been used to develop various other methods for epigenetic analysis to look at DNA-protein interaction and protein modifications including those present in histone proteins. Chromatin immunoprecipitation (ChIP) can be used to identify protein modifications and DNA-protein interactions. It involves *in vivo* cross-linking of proteins to DNA with formaldehyde and then isolation of crosslinked chromatin from nuclei. The isolated chromatin is further sheared into smaller fragments followed by immunoprecipitation with the help of specific antibodies. The isolated DNA with this procedure can be investigated with specific primers or combined with high throughput NGS creating powerful tools like ChIP-Seq (Diaz et al., 2017). Various useful modifications like sequential ChIP where QPCR and ChIP are used in combination to assess the modifications present on histone proteins H3 and H4 on the target location (Tollefsbol, 2011) and ChlP-chip/ChlP-microarray have been added. Coupled with whole genome microarrays, ChlPs allow the determination of all DNA-binding sites of any protein.

The latest innovations in technology development are anticipated to change the dynamics of DNA methylation research investigations in the future. Efforts have been undertaken to develop methods to study epigenomes where high-quality reference genome sequences are not available. One such transformational technology launched recently (April 2022) is Sequel II/IIe Platform which includes methylation calling in native DNA (MCND) at the same time sequencing is done (https://www.pacb.com). Moreover, the expression of small RNAs can be studied through RNA-seq, RT-qPCRs northern blot, and *in situ* hybridization (Wang Z et al., 2009). All these techniques enable the study of expression and identification of small RNAs and their target predictions. Databases of ncRNAs sequences and their functional annotation from plants like CANTATAdb 2.01 (Szcześniak et al., 2016) with over 200,000 lncRNAs from 39 plant species, MiRbase and TAIR (Rhee et al., 2003; Kozomara et al., 2019) have been very important resources.

## Conclusion and future perspectives

Diverse studies on natural populations of plants including investigations in 1) species from various ecological communities, 2) model and non-model species, 3) species of contrasting habits, 4) species from extreme habitats, 5) species from varied climatic gradients, 6) species with asexual reproduction, and 7) invasive taxa have empirically pointed out the involvement of epigenetic phenomenon in facilitating adaptative ability in plants to cope with the changing environments. Epigenetic mechanisms, either individually or in accompaniment with genetic variation, represent long-term stress responses as compared to transient metabolic and physiological modifications. While short-term responses ensure survival, long-term genetic and epigenetic changes contribute toward plastic and adaptive responses of evolutionary significance. The incidence of phenotypic plasticity in genetically homogeneous populations strongly supports its epigenetic ontogeny, although the causal relationship between the stresses and plasticity still remains to be deciphered. The ability to modulate gene expression to produce stable and heritable alternate phenotypes that affect reproductive success strongly suggests the role of epigenetics in developing adaptability in natural populations in changing and stressful climates. Furthermore, epiallelic transgenerational diversity resulting in novel phenotypes seems to play a major role in helping plants to adapt to new environments by aiding them in expanding their habitat range.

To sum up, evidence indicates that 1) epigenetic marks lead to phenotypic changes in plants and serve as important drivers to rapidly widen the total phenotypic plasticity and phenotypic variance of natural populations, 2) plant epigenomes show high diversity and rapidly generate responses to external stimuli, 3) epigenetic mechanisms help to provide the required variability for selection and expand the plants’ repertoire of adaptive responses 4) epi-variability creates epialleles, that serve as an additional tier of diversity for adaptive evolution 5) epigenetic variability is amenable to selection and may eventually become heritable, 6) epigenetic and genetic fields may complement each other for better execution of phenotypic plasticity and adaptive responses and 7) various biological processes, ecological factors and habitats strongly influence the epigenetic regulatory processes in natural populations (Robertson and Wolf 2012; Trucchi et al., 2016; Amaral et al., 2020; Ashapkin et al., 2020; Ashe et al., 2021; Baduel et al., 2021; Boquete et al., 2021; Qiu et al., 2021; García–García et al., 2022). It has been shown that both genetic and epigenetic variations contribute to environmental stress-induced heritable phenotypic divergence. Many studies have shown higher epigenetic variability than genetic variation (Medrano et al., 2014; Foust et al., 2016; Guo et al., 2018; Wang et al., 2020). Although the population-level studies of epigenetic variability in species with high genetic diversity distributed across different habitats are limited (Robertson et al., 2017; Qiu et al., 2021), future studies in such species would further enhance our knowledge of the interactions between genetic and epigenetic domains of the genome that coordinate adaptive responses.
